# In Silico Investigation of the New UK (B.1.1.7) and South African (501Y.V2) SARS-CoV-2 Variants with a Focus at the ACE2–Spike RBD Interface

**DOI:** 10.3390/ijms22041695

**Published:** 2021-02-08

**Authors:** Bruno O. Villoutreix, Vincent Calvez, Anne-Geneviève Marcelin, Abdel-Majid Khatib

**Affiliations:** 1 Integrative Computational Pharmacology and Data Mining, INSERM UMR 1141, NeuroDiderot, Robert-Debré Hospital, 75019 Paris, France; 2 Sorbonne Université, INSERM 1136, Institut Pierre Louis d’Epidémiologie et de Santé Publique, AP-HP, Hôpitaux Universitaires Pitié-Salpêtrière-Charles Foix, Laboratoire de Virologie, F75013 Paris, France; vincent.calvez@aphp.fr (V.C.); anne-genevieve.marcelin@aphp.fr (A.-G.M.); 3 Université de Bordeaux, INSERM, LAMC, U1029, F-33600 Pessac, France; 4 Institut Bergonié, 33000 Bordeaux, France

**Keywords:** COVID-19, in silico stability prediction, SARS-CoV-2, UK and South African variants, ACE2, spike

## Abstract

SARS-CoV-2 exploits angiotensin-converting enzyme 2 (ACE2) as a receptor to invade cells. It has been reported that the UK and South African strains may have higher transmission capabilities, eventually in part due to amino acid substitutions on the SARS-CoV-2 Spike protein. The pathogenicity seems modified but is still under investigation. Here we used the experimental structure of the Spike RBD domain co-crystallized with part of the ACE2 receptor, several in silico methods and numerous experimental data reported recently to analyze the possible impacts of three amino acid replacements (Spike K417N, E484K, N501Y) with regard to ACE2 binding. We found that the N501Y replacement in this region of the interface (present in both the UK and South African strains) should be favorable for the interaction with ACE2, while the K417N and E484K substitutions (South African strain) would seem neutral or even unfavorable. It is unclear if the N501Y substitution in the South African strain could counterbalance the K417N and E484K Spike replacements with regard to ACE2 binding. Our finding suggests that the UK strain should have higher affinity toward ACE2 and therefore likely increased transmissibility and possibly pathogenicity. If indeed the South African strain has a high transmission level, this could be due to the N501Y replacement and/or to substitutions in regions located outside the direct Spike–ACE2 interface but not so much to the K417N and E484K replacements. Yet, it should be noted that amino acid changes at Spike position 484 can lead to viral escape from neutralizing antibodies. Further, these amino acid substitutions do not seem to induce major structural changes in this region of the Spike protein. This structure–function study allows us to rationalize some observations made for the UK strain but raises questions for the South African strain.

## 1. Introduction

**Figure 1:**
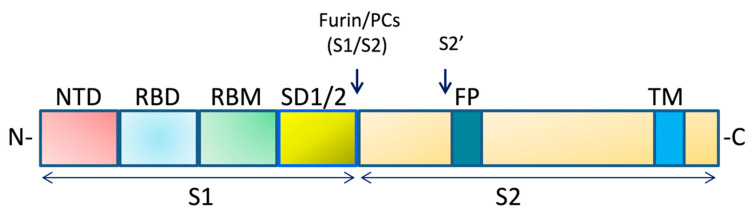
Schematic domain representation of the Spike glycoprotein. The diagram includes the functional domains in the S1 subunit (NTD, N-terminal domain; RBD, receptor-binding domain; RBM, receptor-binding; SD1/2: subdomain 1 and 2) and in the S2 subunit (FP, fusion peptide; TM, transmembrane domain. The N- and C-terminal domains are indicated. Arrows denote the protease cleavage sites. PCs: Proprotein convertases.

Since the occurrence of the Coronavirus Disease 2019 (COVID-19), a virulent disease mediated by SARS-CoV-2 initially identified in China, COVID-19 has caused 2,238,022 deaths with 103,538,277 cases as visualized on 1 February 2021 (daily online worldwide data about COVID-19: https://www.worldometers.info/coronavirus/ (accessed on 25 January 2021)). Although the pathogenesis of this disease remains unclear, in addition to the host response mediated by SARS-CoV-2, variations in the viral strains seem to also be involved in differences in transmission/infectivity and/or severity of the disease. SARS-CoV-2 is a large, enveloped, single-stranded positive-strand RNA virus containing four major structural proteins, namely Spike (S) protein, Nucleocapsid (N) protein, Envelope (E) protein, and Membrane (M) protein. The N protein with multifunction is involved in virus replication, transcription, and assembly and physically interacts with the viral membrane protein during virion assembly [1]. The S protein is a large oligomeric transmembrane protein that mediates virus entry into host cells. The S protein is composed of two subunits, namely S1, responsible for receptor binding, and S2, which mediates downstream membrane fusion (Figure 1) [2,3].

**Figure 2:**
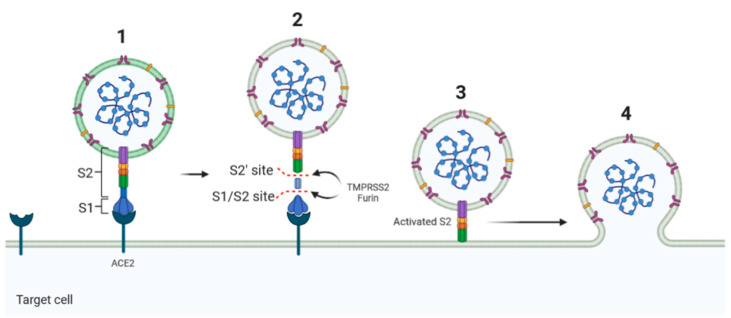
Schematic diagram of the Spike–ACE2 interaction. SARS-CoV-2 exploits the angiotensin-converting enzyme 2 (ACE2) to enter target cells. After receptor binding (1), the virus S protein is cleaved by proteases such as furin/TMPRSS2 into S1 and S2 subunits (2) that mediates S2-assisted fusion (3) and the release of the viral genome (4).

For entry into target cells, SARS-CoV-2 exploits the angiotensin-converting enzyme 2 (ACE2) as a receptor (Figure 2) [4]. Therefore, the S protein determines in part the infectivity of the virus and its transmissibility in the host [5]. Indeed, a small, isolated folded domain of the S1 subunit was reported as the receptor-binding domain (RBD), directly interacts and binds ACE2 during the virus contact with the target cell [6]. This interaction occurs following S protein cleavage at the S1–S2 junction by a furin-like pro-protein convertase (PCs) expressed by the host cells [6]. The S proteins are further processed in the target cells within the S2 domain at the S2ʹ site, a process that is also necessary for efficient infection [7].

Viruses continually change through mutations, a mechanism responsible for the emergence of new variants. Particularly, RNA viruses have been reported to have elevated mutation levels as compared to DNA viruses [8,9]. In the surface protein of various viruses such as Ebola virus [10], Chikungunya virus [11] and the highly pathogenic avian influenza H5N1 [12], amino acid changes were found to significantly alter viral functions, often resulting in an increased transmissibility and/or mortality. 

In the gene encoding the S protein of SARS-CoV-2, various mutations have been reported [13,14] and recently, the United Kingdom (UK) and South Africa have faced a rapid increase in COVID-19 mediated by new variants that are named in the literature (VOC-202012/01 or VUI-202012/01 or B.1.1.7 for UK and 501Y.V2 or 20C/501Y.V2B.1.351 for South Africa). These variants harbor one or several nonsynonymous spike mutations including amino acid replacements at key sites in the spike RBD domain (K417N, E484K, N501Y for the African strain [15] and only N501Y in this region of the protein for the UK strain [16]). The proportion of these variants has increased rapidly and recent observations suggest that they are significantly more transmissible than previously circulating variants. However, it is still not fully known if the pathogenicity is increased, although some elements have been recently released for the UK strain with likely enhanced disease severity [17]. Furthermore, at present, it is still unclear why some individuals are more susceptible to virus infection and what could be the role of mutations on ACE2 or on the Spike protein. 

We here attempt to gain novel insights over this protein–protein interaction using various in silico approaches while integrating previously reported experimental data. We specifically focus on amino acid changes found in the South African and UK strains located in the Spike RBD domain with a special emphasis on the potential impact of the only three residues (K417, E484 and N501) that are located directly at the interface with ACE2.

## 2. Results 

### 2.1. Analysis of the Spike-RBD “Unbound” State

**Figure 3:**
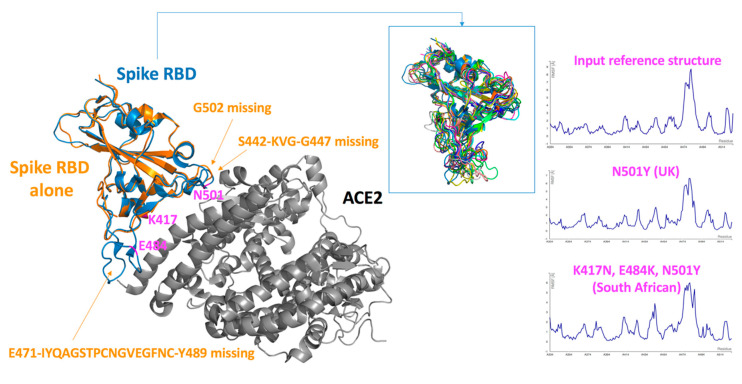
Analysis of the Spike receptor-binding domain (RBD) in a simulated unbound state. (Left) The Spike RBD domain as seen in complex with ACE2 (in grey) is shown in blue (PDB entry 6M0J). The Spike RBD 3D structure as defined in the absence of the ACE2 receptor (PDB entry 6VYB) is shown in orange. The amino acids of interest for this study are in magenta. It is seen that some loops are not fully defined in 3D when the Spike protein is crystallized in the absence of the ACE2. These loops should thus be relatively flexible in solution. As residues 501 and 484 are located in these peptide segments, it was important to predict the possible structural changes in the RBD in the unbound state. The 3D structure of the RBD as found in the PDB entry 6M0J was selected (the ACE2 atoms were deleted) and used as input for the simulations. In the inset, middle of the figure, one can see ten representative structures of the simulated nonmutated RBD input structure (there are no major differences when visualizing the variants). All the models are superimposed on the input structure (cartoon diagram, painted in blue) and then each model has a different color. One notices that some loops are flexible, the most important structural changes when removing the ACE2 structure occur in the loop involving residues 471 to 489. This loop should adopt several conformations in solution, and be stabilized upon binding to the ACE2 (and most likely when interacting with some antibodies). (Right) The fluctuation plots (RMSF in Å) obtained for the Spike RBD without substitution, the N501Y variant and the K417N, E484K, N501Y variant are shown. These three proteins have the same overall RMSF profiles, with some small changes that differ in the South African strain as compared to the initial input structure or to the UK strain. This suggests that the 3D structures of this domain of the Spike should be resilient to such amino acid changes in term of conformational changes. In addition, we do not expect these replacements to destabilize the unbound Spike protein suggesting that many of the effects seen for these variants could be due to affinity changes toward ACE2 (see text).

Prior to the analysis of the Spike–RBD–ACE2 complex, we investigated the Spike-RBD domain alone. Yet, in the experimental structures of the Spike protein studied without ACE2 (e.g., PDB entry 6VYB, SARS-CoV-2 Spike ectodomain, open state reported by Walls et al., [3]) or in the absence of co-crystallized antibodies, some Spike residues are missing, essentially in three loop regions (i.e., the loop containing G502, the loop containing residues 442–447, loop containing residues 471–489) (Figure 3). This strongly suggests that these segments are flexible and can certainly adopt different conformations in solution. These conformational ensembles should differ from the structure seen in the bound Spike–ACE2 state (e.g., PDB entry 6M0J). In fact, two residues of interest for the present study, namely E484 and N501, are located in these loops. Upon complex formation with ACE2 and/or monoclonal antibodies, these loops should be stabilized and they are then visible in these biophysical experiments. To explore the potential conformational changes due to the amino acid changes in the RBD domain, we used the conformation of the RBD domain cocrystallized with ACE2 (PDB entry 6M0J) but we removed ACE2. The coarse-grained simulations were performed on the initial RBD input structure, on the N501Y variant (UK strain) and on the K417N, E484K, N501Y variants (South African strain). The overall Root Mean Square Fluctuation (RMSF) profile of the initial input structure (the simulated RBD without ACE2) suggests a high flexibility in several loops, specifically in the segment encompassing residues 471–489 (Figure 3). This indicates a structural rearrangement of the Spike protein upon binding. Yet, the overall RMSF profiles of the initial structure and the UK variant are very similar (Figure 3), suggesting that they should have a similar topology. From these computations, it seems that the N501Y does not induce a large conformational change. This is somewhat supported by a recent cryo-EM structure of the N501Y mutant co-crystalized with a potent neutralizing antibody [18]. Of course, the Spike protein is not in an unbound state, yet this structures of the N501 Spike protein with the antibody and of the Y501 variant with the antibody are very similar; in both cases, the backbone atoms of these two residues superimpose very well, indicating that this substitution is unlikely to induce large structural changes. These structural data further suggest that the N501Y variant can still be neutralized efficiently by an antibody, indicating that most likely, vaccines should be efficient against this variant. This would seem consistent with a preliminary report for the Pfizer-BioNTech product [19]. 

If we then compare the overall RMSF profiles of the initial input Spike-RBD structure with the computer simulated South African variant, we note some small differences, but we do not expect major conformational changes, consistent with the fact that this variant binds well to ACE2. It seems reasonable to suggest that antibodies should interact well with this variant (i.e., the conformational changes should not be dramatic), unless of course, the key binding site of the antibodies directly involves the mutated residues (hotspot residues). As such, additional experimental data are needed to fully evaluate the efficiency of the vaccines against this South African strain. 

We also investigated the stability changes upon amino acid replacements and thus computed the possible effects on the folding free energy (∆∆G) using DUET and DynaMut2. As the exact 3D structure of the unbound RBD is not known, we computed these values on ten selected diverse 3D structures obtained from the CABS-Flex simulations. The N501Y (UK) was found to be slightly destabilizing (depending the input simulated structures and methods, around −0.2 to −0.3 kcal/mol). In fact, such small changes are not significant and suggest that this Spike protein variant is not destabilized. This is also consistent with the previously reported experimental data [20] as this variant is expressed at the same level as the reference structure. The combined K417N, E484K, N501Y substitutions also do not seem to destabilize the RBD domain of the Spike protein as for all the ten simulated structures; we obtained ∆∆G values around −0.5 or −0.6 kcal/mol. Here again, such values suggest that the variant protein is not destabilized due to these three amino acid changes. In fact, experimentally, it was found that the expression level of each three single substitutions was similar to that of the reference protein without these point mutations [20]. The triple mutant was not tested but our computations suggest that the protein is resilient to such amino acid changes in these locations.

### 2.2. Analysis of the Spike-RBD-ACE2 Complex

The crystal structure of the Spike–RBD–ACE2 complex was analyzed using COCOMAPS and the results of the analysis indicate that 52 residues are involved in the interaction from the ACE2 side while 46 residues (see below) in the Spike RBD could play a role at the interface. About 1688 Å^2^ is buried upon complex formation (total surface area buried from both proteins) and the buried polar surface represents about 58% of the interface while the nonpolar buried surface involves about 42% of the interface. A variety of noncovalent interactions are found between the amino acids of both the Spike protein and the ACE2 receptor. The identified residues on the Spike protein that have some contacts (direct or indirect) with ACE2 (within a maximum distance of about 8 Å from the ACE2 interface) involve the following 46 amino acids: R403, D405, E406, K417, Y421, N439, K444, V445, G446, G447, N448, Y449, Y453, R454, L455, F456, R457, Y473, Q474, A475, G476, S477, T478, E484, G485, F486, N487, C488, Y489, F490, P491, L492, Q493, S494, Y495, G496, F497, Q498, P499, T500, N501, G502, V503, G504, Y505, Q506 (here the strength or energetics of the interaction is not considered, see below).

**Figure 4:**
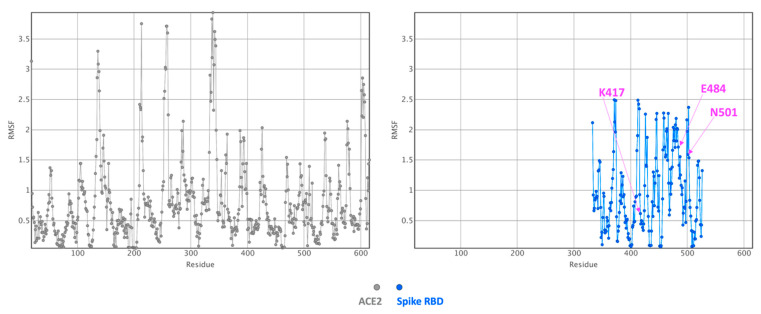
Fluctuation plot obtained for the Spike RBD–ACE2 complex. The residue fluctuation profile (RMSF in Å) as computed with CABS-Flex is shown (ACE2, in grey and the Spike domain, in blue). Substituted residues in this region of the Spike protein in the UK strain (N501) and in the South African strain (K417N, E484K, N501Y) are labelled in magenta.

The flexibility of the Spike RBD–ACE2 complex was then investigated with CABS-Flex. The complex was simulated using the crystal structure as the starting point (Figure 4). 

The main information that we obtain here is that many segments at the interface are relatively rigid, as expected, when the two proteins are bound. Yet, a peptide loop at the interface, involving the Spike protein residues 475 and 487 is predicted to be more flexible than the other regions at the interface. This is the same loop that we found very flexible in the computations reported above. Yet, here the RMSF values are significantly lower than for the RBD alone, indicating stabilization upon complex formation. Some other segments on both proteins (ACE2 and Spike protein) located relatively far away from the interface (essentially loops) are also predicted to be flexible. Simulations of the variant models do provide similar observations (i.e., the substitutions did not enhance or significantly reduce the flexibility or rigidity of the interface: data not shown).

We then investigated the global binding scores for the original Spike–ACE2 structure as present in the PDB file (PDB entry 6M0J) and for the UK and South African strains with the SPServer. The PAIR score gives some insights about the energetics of the interaction. It was found to be more favorable for the UK strain (N501Y) as compared to the initial structure (i.e., when N is at position 501). Yet, the predicted global interaction score between the Spike and ACE2 proteins seems less favorable for the South African strain (K417N, E484K, N501Y) than for the original input structure or for the UK strain. 

**Figure 5:**
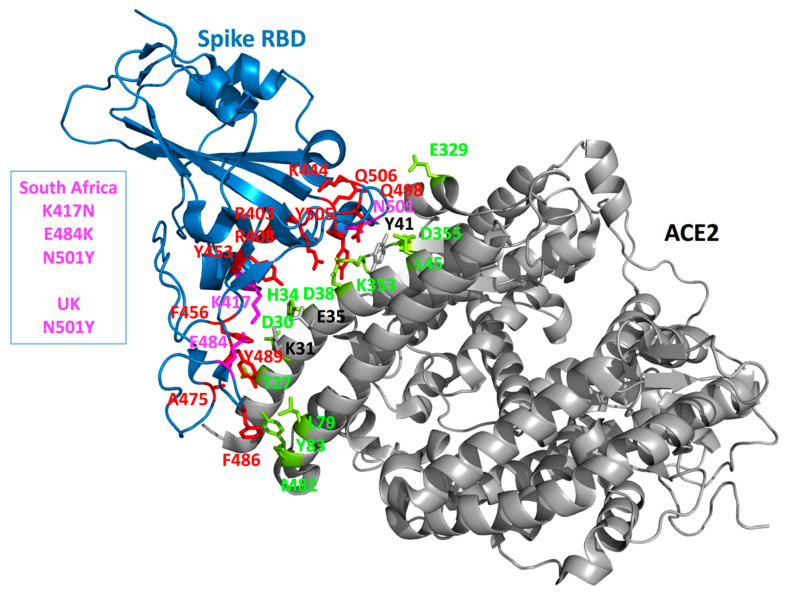
Mapping key amino acids at the Spike–ACE2 interface. The crystal structure of the Spike RBD and part of the ACE2 receptor interacting with the Spike is shown as cartoon diagram (PDB entry 6M0J). The Spike domain is in blue and the ACE2 domain in grey. The Spike side chains predicted to be energetically important (with our in silico protocol, see the Method section) for the interaction with ACE2 are shown in red. The UK and/or South African strains are highlighted by coloring the amino acids in magenta. All the Spike side chains shown here should have favorable interaction energies with ACE2 but E484 that is predicted to have very weak or unfavorable interactions. On the ACE2 side, the side chains that are predicted to contribute favorably to the interaction with the Spike RBD are shown in lemon yellow. Only ACE Y41 (in grey) seems to have very limited interactions with the Spike protein while, when the Spike protein carries a Y at position 501, ACE2 Y41 has then some favorable interaction energy values with the Spike. Two other ACE2 residues can be mentioned here, K31 and E35 (in grey, see Text).

In a following step, we predicted the interaction energy of the Spike RBD–ACE2 protein–protein complex partitioned at the residue level using pyDockEneRes. On the Spike RBD domain, the top residues that are predicted to have favorable interaction energy values with the region of ACE2 that binds the RBD are (from the most favorable according to this method, values of about −12 kcal/mol to less favorable but still with some contributions around −1 kcal/mol): F486, F456, Y505, Y489, R403, K417, Y453, R408, K444, N501, Q498, Q506, and A475 (Figure 5). Some other residues are present at the interface on the Spike protein but the computed interaction energy values were outside our selected range of energy values (e.g., Spike Q493, Y449, L455, G485, or G496). Of interest, in the list of residues predicted to be important for the interaction with ACE2, Spike N501 (present in both the UK and South African strains) and Spike K417 (South African strain) were identified while E484 (the third substitution in this region of the Spike protein in the South African strain) was not predicted to be a major residue for this interaction. On ACE2, the top key predicted residues involved (from very favorable energy values around −7.7 kcal/mol to less favorable, around −1 kcal/mol) are: T27, Y83, K353, M82, D30, H34, D38, D355, E329, L45, and L79 (Figure 5). Here also, a few extra residues are present at the interface (like ACE2 Y41, K31, E35) but the computed energy values were too weak considering the selected threshold value of about −1 kcal/mol. 

If we then consider the three residue replacements of interest here (N501Y, K417N, E484K), we observe that N501 (UK and South African strains) is solvent exposed on the free Spike protein and becomes essentially buried upon ACE2 binding. N501 is located in a loop structure and could be replaced by a Y without creating folding problems. Some flexibility is present there suggesting that a larger Y residue could be accommodated (Figure 4). Some weak hydrogen bonds (i.e., the distances are too long to form strong hydrogen bonds as the distances in some are cases close to 4 Å) are possible between Spike N501 and ACE2 K353 (a residue found to have some contributions with the pyDockEneRes tool) and ACE2 Y41 (not listed in our analysis as the computed contribution with this method is too weak considering our energy threshold) (Figure 5). The contribution of N501 to the interaction with ACE2 is predicted to be around −1.6 kcal/mol but when a Y is present at this position, the variant residue has a predicted interaction energy with ACE2 of about −3.5 kcal/mol. On the graphics display, we note that the newly introduced Y501 side chain should make many new favorable nonbonded interactions with ACE2, like for example with ACE2 Y41 via a Pi-stacking, with ACE2 K353 via a cation-Pi interaction and possibly with ACE2 D38 via a hydrogen bond. These types of noncovalent interactions (e.g., Pi-stacking and cation-Pi) in the variant should be very favorable for the interaction and much stronger as compared to the initial N residue. It is important to note that such energetics terms are in general underestimated and/or not considered by many scoring functions as very difficult to calibrate.

**Figure 6:**
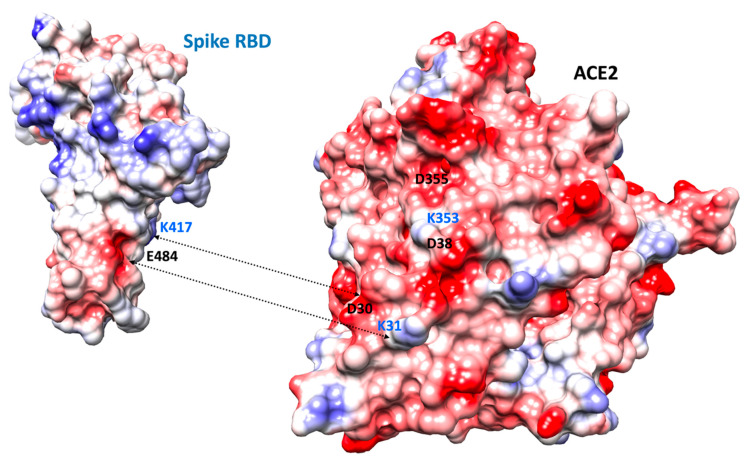
Molecular surface of the Spike and ACE2 proteins color-coded according to electrostatic potentials. The potentials were computed using Coulomb’s law. The orientation is slightly modified as compared to Figure 5 and the molecules were spread apart so as to better see the interface. Some residues are labelled for orientation. The color gradient runs from electronegative values (−10 kcal/(mol.e) in red), to more neutral (white) to electropositive (+10 kcal/(mol.e) in blue). The interface area on ACE2 tends to be electronegative while for the Spike protein, we observe two regions, one electronegative and one electropositive, separated by a more neutral zone.

Spike K417 is located at the N-term of a short helix, it is solvent exposed and becomes partially buried upon binding to ACE2. It is in a somewhat rigid region of the interface (Figure 4, Figure 5 and Figure 6). It can however be replaced by N (South African strain) without creating folding problems and as mentioned above, the substitution should not destabilize the Spike protein. K417 forms a salt-bridge with ACE2 D30 and should contribute favorably to the interaction as its predicted energy value is around −3 kcal/mol. Upon replacement by a N, the contribution of position 417 to the interaction with ACE2 is less favorable than with the K (loss of a salt-bridge) and is computed to be −1.3 kcal/mol. Some weak hydrogen bonds could still be possible, most likely with ACE2 H34 and D30.

E484 is located in a loop structure and is solvent exposed. This segment is somewhat more flexible than the remaining regions of the interface (Figure 4, Figure 5 and Figure 6). It remains solvent exposed upon binding to the ACE2 protein. Its contribution to the interaction energy is predicted to be relatively weak despite the presence of the nearby ACE2 K31 (which forms a salt-bridge with ACE2 E35). The computed interaction energy between these two residues was not significant, most likely because the distance between the two opposite charge centers is around 4.4 Å and thus slightly higher than 4 Å commonly found in energetically stronger salt-bridges (i.e., strong salt bridges usually require opposite charges to be around 4 Å or below) and as the interaction is fully solvent exposed. E484 could be replaced by a K in the Spike protein without creating folding problems (see also the section above). Yet, a positively charged K at this position in the ACE2–Spike complex is predicted to be less favorable than a negatively charged E residue with regard to the interaction with ACE2 (about +1.9 kcal/mol, a positive Spike K484 could have unfavorable interaction with a positively charged ACE2 K31). 

## 3. Discussion

Amino acid changes in a protein can have numerous impacts, from folding problems to the modulation of intermolecular interactions with ligands or protein partners. Several key structural properties are known to play a role here, such as the type of amino acid substitution (conservative replacement or not), the location in the protein structure (e.g., the change takes place in a loop or in secondary structure elements, the residue is buried or solvent exposed), the residue is inside a catalytic site or within a protein-ligand interface, the replaced residue is in a flexible or rigid region among many others [21,22,23,24,25,26,27,28]. Different types of computational tools (https://www.vls3d.com/ (accessed on 25 January 2021)) [22,29], all with strengths and weaknesses, can be used to investigate the possible impacts of amino acid replacements on the structure and function of a protein when a 3D structure is available or can be predicted [21,22,23,24,25,26,27,28]. It is recommended to use different tools that apply different types of algorithms so as to gain some “consensus” insights about the amino acid replacement [21]. Further, when experimental data are available, it is obviously of interest to select methods that can at least reproduce such data. The present investigation benefits from the recently published Spike protein mutagenesis study and the impacts on ACE2 binding [20]. Among the experimental data reported in that study, we were particularly interested in the measured affinity of the Spike N501F protein mutant with ACE2 as it involves residue 501 and as the affinity was assessed in a purified binding assay (most of these other affinity evaluations were performed using high-throughput affinity measurements that could be less accurate than measurements carried out in purified systems). Experimentally, it was found that the Spike N501F protein has increased affinity for ACE2. Interestingly, most in silico tools that we tested to compute stability changes at the protein interfaces (ΔΔG) could not reproduce this measurement (data not shown). We noted, however, that the SPServer and pyDockEneRes tools were able to output values consistent with this high-quality N501F experimental result. Our selection of tools does not mean that these in silico approaches will systematically outperform the other methods in all cases but that they are valuable for this specific study. Further, while these tools have been assessed extensively by the authors, we still decided to compare the results of these two methods on five protein–protein interactions selected from the SKEMPI database [30] (thus with measured experimental stability changes upon mutations) to gain higher confidence in the values of the computed scores. That is, in general, all published methods that predict stability changes at the interface are compared with experimental values and correlations between the predictions and the experimentally measured values are reported. This is of importance but equally important is to gain insights over the type of substitutions that are well-addressed by the methods. For example, some methods may or may not recognize a salt-bridge depending on the implemented parameters [21]. Furthermore, the energetics (on a single protein or at the interface) of several selected amino acid changes should make sense as compared to what is seen on the computer display.

Several crystal structures of the ACE2 and SARS-CoV-2 receptor-binding domain (RBD) have been reported (e.g., PDB IDs: 6LZG, 6M17, 6M0J) [31,32,33] and help the investigation of residues at the interface. Before discussing further amino acid changes in the RBD domain of Spike, it is interesting to briefly review some structure–function data reported on polymorphisms in the ACE2 gene, as some have been proposed to reduce the affinity toward the Spike protein, with possibly some subsequent lower susceptibility to infection [34]. For example, structural and in silico analyses of ACE2 polymorphisms have been carried out on the ACE2 region that directly interacts with the Spike glycoprotein [35]. In that study, the authors found that two substitutions in ACE2 positions 19 and 26 could modulate the affinity for the Spike protein (ACE2 position 19, fully solvent exposed—the S19P is common in African people and the substitution was suggested to protect individuals (reduced affinity for Spike) and a cleavage site of the ACE2 precursor; ACE2 position 26, fully solvent exposed—the K26R is common in European people, the mutation may increase affinity of ACE2 towards Spike). In our hands, it would seem that the ACE2 K26R may only have minor roles with regard to Spike binding. Indeed, in our investigation, ACE2 residue K26 was not found to be a major player that strongly contributes to the stability of the interface because it is at about 8 Å from the Spike protein in all the available experimental structures of the complex and as it tends to point away from the Spike surface (of course some side chain flexibility is likely but in the available X-ray structures, around ACE2 K26 we note that the closest residue is the Spike N487 side chain, at about 7.8 Å, much too far for hydrogen bonds while the Spike K417 side chain is at about 7 Å, leading most likely to small electrostatic charge–charge repulsion). Some long distance electrostatic interactions may play a role in the preorientation of the two binding partners and be more favorable when ACE2 K26 is replaced by arginine. Another most likely alternative could be that, as ACE2 K26 makes a salt-bridge with ACE2 E22 and polar interactions with ACE2 N90, the K26R substitution stabilizes locally this region of ACE2 (possibly creating another salt-bridge with the nearby ACE2 E23, not possible when ACE2 at position 26 is a K) and this could contribute to a more favorable change in free energy of binding with the Spike protein. Additional investigations are needed to clarify this point. Other studies investigating genetic variations in ACE2 suggest that amino acid changes in regions that are relatively far away from the interaction site for the Spike protein could also alter the recognition via various molecular mechanisms [36].

For the Spike protein, some previously reported mutations have also been analyzed and it was found that some could increase the transmissibility and/or pathogenicity of the virus. For example, the Spike D614G substitution [14,37] seems to increase the affinity with the ACE2 receptor although this residue is not present at the Spike–ACE2 interface but at a Spike protomer–protomer interface. However, here we are more specifically interested in the analysis of three amino acid substitutions found in the UK strain and/or in the South African strain, with a special emphasis on residues that are directly present on the Spike receptor-binding domain, at the interface with ACE2. We wanted to gain some insights about the putative impacts of these substitutions on the interaction with the ACE2 receptor. Our in silico results were also analyzed in the light of recently reported experimental mutagenesis data [20]. In fact, experimentally, amino acid can be substituted and the expression levels monitored (e.g., notion of stability problem if, for instance, the mutant protein is expressed at very low level). Such behavior can also be estimated in silico in some cases. Further, when a mutant protein is expressed, its interaction with a protein partner can be evaluated. This event can also be investigated to some extent in silico. In all cases, it is important to note that both experimental data and in silico predictions can be misleading and as such results should be investigated with cautions.

In the previously reported Spike protein mutagenesis study mentioned above [20], it was found that the replacement of the Spike protein residue N501 by an F enhances ACE2 affinity while having no effects on expression. This observation is expected for the N501Y substitution although the measurements were not performed in purified system. Experimental data for the Spike protein residue 501 are in agreement with the present computer analysis. The replacement of K417 by N (South African strain) seems relatively neutral in term of protein expression level while not very favorable for the interaction with ACE2 [20], also in agreement with our computer evaluation. The E484K replacement was not found to change the expression level, suggesting, as observed in our structural analysis, that a K could be present in this position of the Spike protein without creating major structural changes. Yet, the substitution with a K seems experimentally to slightly enhance the affinity towards the ACE2 receptor, while we computed that a K at this position should be less favorable than an E. The differences between the computed values and the experimental observation could be due to inaccuracies in the scoring functions and the unexpected flexibility at the interface. It is also important to note that the substitutions in the South African and UK strains outside the interface area with ACE2 could play a role in affinity and as such on transmission/infectivity. Yet, when focusing only on the Spike RBD–ACE2 interface, the present in silico predictions and interactive structural analysis suggest that the key player residue in term of enhanced affinity is the N501Y replacement. Our observation applies essentially to the UK strain while additional work is required to gain insights into the South African strain because in our hands, looking only at the interface, it would seem that the K417N and E484K substitutions are not favorable for the interaction with ACE2. For the time being, we do not know if the N501Y alone could counterbalance the predicted unfavorable Spike K417N and E484K amino acid replacements. Considering the South African strain, it does not seem that the Spike RBD has higher affinity for ACE2 as compared to the reference Spike. If indeed the transmission is enhanced (of course transmission involves complex mechanisms), this could be due to mutation outside the direct ACE2–Spike interface. If the pathogenicity of the South African strain is higher than the reference strain, this could be due in part to an amino acid change at residue 484 which upon substitution seems to impede recognition by neutralizing antibodies [38,39]. For the UK strain, it is possible that the enhanced affinity to ACE2 due to the N501Y substitution increases transmissibility but also contributes to disease severity (not only due to the increased number of infected individuals) as suggested after comparisons of the SARS-CoV-2 with other human coronaviruses [40]. From the simulations performed here it seems that the N501Y substitution or the N501Y, K417N, E484K replacements should not induce major conformational changes, suggesting that the newly developed vaccines should be efficient against these variants. Yet, for the South African strain, the substitution at residue 484 (located in a loop protruding at the surface) could potentially create minor problems.

## 4. Materials and Methods

We used the crystal structure of the Spike–RBD–ACE2 complex (PDB entry 6M0J) reported by Lan et al. [31] to map amino acid changes of the African and UK strains in 3D. We took into account the results of deep experimental mutational scanning of the Spike RBD [20] to select the in silico tools that will be used to compute the energetics of the interaction and during the interactive structural analysis. COCOMAPS was used to analyze contacts at the biomolecular interface [41]. Flexibility of the Spike RBD domain alone and the ACE2–Spike complex were investigated with the CABS-Flex package [42,43]. Computations were performed on the initial structures (reference sequence) and on the variants generated by computer means (see below). The applied simulation approach uses a coarse-grained protein model to investigate flexibility so as to speed up the computations as compared to classical all-atom molecular dynamics and was shown to provide a similar overview of the system. Restraints were applied to take into account disulfide bonds present in the structure. The simulations were repeated 10 times each.

The global interaction energy differences between the initial Spike–ACE2 structure, the UK and South African strains were investigated with the SPServer [44]. This analysis uses split-statistical potentials to for instance investigate the interaction energy differences between native and mutant structures. Split-statistical potentials are knowledge-based potentials that consider the frequency of pairs of residues in contact, the nature of the amino acids and can include information about structural environment. In our computations, a Cbeta-Cbeta distance <12 Å was used to instigate residues in contact. Different types of scores are reported. For instance, the score named PAIR is obtained by summing the potential of mean force with the corresponding subindex of each pair of interacting residues a, b, between the Spike and ACE2 proteins. It considers amino acid frequencies along distances. The pyDockEneRes software uses a different scoring function; it was used to provide interaction energy values of the protein–protein complex partitioned at the residue level [45]. 

Interactive structural analyses were performed with PyMol (Schrödinger product, New York, NY 10036-4041, USA) and UCSF ChimeraX (University of California, San Francisco, CA 94158-2517, USA) [46]. Amino acid substitutions in the Spike protein were introduced using PyMol while a short energy minimization of each modified 3D complex was carried out with Chimera. Different low energy rotamers were investigated whenever appropriate. Possible effects on the folding free energy (∆∆G) due to the amino acid changes were investigated on ten representative 3D structures of the Spike RBD models after simulations performed in the absence of the ACE2 structure. These computations were performed on each 3D structure using DUET (which combines the results of two different computational approaches) [47] and DynaMut2 [48], an approach that can assess changes in stability upon single and multiple missense mutations and integrate Normal Mode Analysis during the computations. Electrostatic properties were computed using several routines implemented in the Chimera package.
